# Multimodal Approach for Emotion Recognition Based on Simulated Flight Experiments

**DOI:** 10.3390/s19245516

**Published:** 2019-12-13

**Authors:** Válber César Cavalcanti Roza, Octavian Adrian Postolache

**Affiliations:** 1 Instituto Universitário de Lisboa (ISCTE-IUL) and Instituto de Telecomunicações (IT-IUL), Av. das Forças Armadas, 1649-026 Lisbon, Portugal; 2 Universidade Federal do Rio Grande do Norte (UFRN), Av. Sen. Salgado Filho, 3000, Candelária, Natal, RN 59064-741, Brazil

**Keywords:** emotion recognition, physiological sensing, multimodal sensing, deep learning, flight simulation

## Abstract

The present work tries to fill part of the gap regarding the pilots’ emotions and their bio-reactions during some flight procedures such as, takeoff, climbing, cruising, descent, initial approach, final approach and landing. A sensing architecture and a set of experiments were developed, associating it to several simulated flights ($N_{flights}=13$) using the Microsoft Flight Simulator Steam Edition (FSX-SE). The approach was carried out with eight beginner users on the flight simulator ($N_{pilots}=8$). It is shown that it is possible to recognize emotions from different pilots in flight, combining their present and previous emotions. The cardiac system based on Heart Rate (HR), Galvanic Skin Response (GSR) and Electroencephalography (EEG), were used to extract emotions, as well as the intensities of emotions detected from the pilot face. We also considered five main emotions: happy, sad, angry, surprise and scared. The emotion recognition is based on Artificial Neural Networks and Deep Learning techniques. The Root Mean Squared Error (RMSE) and Mean Absolute Error (MAE) were the main methods used to measure the quality of the regression output models. The tests of the produced output models showed that the lowest recognition errors were reached when all data were considered or when the GSR datasets were omitted from the model training. It also showed that the emotion *surprised* was the easiest to recognize, having a mean RMSE of 0.13 and mean MAE of 0.01; while the emotion *sad* was the hardest to recognize, having a mean RMSE of 0.82 and mean MAE of 0.08. When we considered only the higher emotion intensities by time, the most matches accuracies were between 55% and 100%.

## 1. Introduction

With the growth of air safety and accident prevention, especially in the mechanical–structural and avionics aspects, a gap of probable cause of accidents is emerging, which can justify the occurrence of several unwanted situations. This can be referred to as the relationship between emotions and aviation accidents caused by human failure.

The development of research about the relation between emotions and aviation activities is quite new and is mainly based on preliminary and final accident reports. It was important to show the real need of improvements and strategies regarding emotion effects in risky situations of a real flight, mainly on take off, approach and landing.

To know how important are the studies of emotions over the aviation contexts, we first need to understand emotion definitions. Emotion is led by the brain and it can sometimes be the result of chemical processes that join several internal and external factors to produce an output or response that reflects an emotional state [1]. This response can also reflects some physiological changes in our human body [2]. Some emotions e.g., the primary emotion "anger" plays a fundamental role in many cases, such as fear and trust, that are directly related to protection, defense and maintenance of life.

Several methods and techniques can be applied to perform emotion recognition through the use of a couple of hardware devices and software such as: analysis of emotional properties based on two physiological data such as, ECG and EEG [3]; unified system for efficient discrimination of positive and negative emotions based on EEG data [4]; automatic recognizer of the facial expression around the eyes and forehead based on Electrooculography (EOG) data giving support to emotion recognition task [5]; use of GSR and ECG data to develop a study to examine the effectiveness of Matching Pursuit (MP) algorithm in emotion recognition, using mainly PCA to reduce the features dimensionality and Probabilistic Neural Network (PNN) as the recognition technique [6]; emotion recognition system based on physiological data using ECG and respiration (RSP) data, recorded simultaneously by a physiological monitoring device based on wearable sensors [7]; emotions recognition using EEG data and also performed an analyze about the impact of positive and negative emotions using SVM and RBF as the recognition methods [8]; new approach to emotion recognition based on EEG and classification method using Artificial Neural Networks (ANN) with features analysis based on Kernel Density Estimation (KDE) [9]; an application that stores several psychophysiological data based on HR, ECG, SpO2 and GSR, that were acquired while the users watched advertisements about smoking campaigns [10]; experiments based on flight simulator to developed a multimodal sensing architecture to recognize emotions using three different techniques for biosignal acquisitions [11]; multimodal sensing system to identify emotions using different acquisition techniques, based on photo presentation methodology [12]; real-time user interface with emotion recognition that depends on the need for skill development to support a change in the interface paradigm to one that is more human centered [13]; recognize emotions through psychophysiological sensing using a multiple-fusion-layer based on ensemble classifier of stacked auto encoder (MESAE) [14].

In addition, it is also possible to present some research that is more related to emotion analysis e.g., the use of the Friedman test to verify whether the work on exposure and emotional identification influences helps to decrease the levels of anxiety and depression [15]; emotion recognition system based on cross-correlation and the Flowsense database [16]; derived features based on bi-spectral analysis for quantification of emotions using a Valence-Arousal emotion model, to get a way of gaining phase information by detecting phase relationships between frequency components and characterization of the non-Gaussian information from EEG data [17]; a novel real-time subject-dependent algorithm using Stability Intra-class Correlation Coefficient (ICC) with the most stable features that gives a better accuracy than other available algorithms when it is crucial to have only one training session [18]; analysis of emotion recognition techniques used in existing systems to enhance ongoing research on the improvement of tutoring adaptation [19]; and the ensemble deep learning framework by integrating multiple stacked auto-encoder with parsimonious structure to reduce the model complexity and improve the recognition accuracy using physiological feature abstractions [20].

In the present work, we mainly studied the multimodal or multisensing architecture, processing, feature extraction and emotion recognition, regarding the pilots’ feelings during a couple of simulated flights. These “pilots” in command, were represented by the beginner users of a flight simulator (not real pilots), following a sequence of steps during the flight experiments. The result of this work can also be applied to several workplaces and contexts e.g., administrative sectors [21], in aviation companies/schools [11] and in urban areas [16], among others.

### Main Motivation and Contribution

Among a broad set of possible applications of the developed sensing architecture, the use of emotion recognition applied to an aviation context was the chosen one.

In 2017, Boeing presented a statistical summary [22], about commercial jet airplane accidents confirmed in worldwide operations for 1959 through 2016. It considered airplanes that were heavier than 60,000 pounds maximum gross weight. There was a very clear statistical analysis, in which it was possible to note the impressive evolution of aviation safety along the past years. As well as Boeing, the International Civil Aviation Organization (ICAO) also presented a similar report considering the period between 2008 and 2018, showing the same evolution of aviation safety along this period [23]. Every year, aviation has become safer, reaching lower levels of accidents with fatalities including hull losses or not. Although, there are no reasons to completely relax, because there are other problems to solve: the psychophysiological aspect inside a real flight operation.

According to several reports from the last 5 years, it is easy to observe that the main causes of these accidents were human failures, which some of it were also associated with human emotions. Based on that, we can note that aviation safety is facing a new age of accident factors i.e., the “age” of aviation accidents caused by human failure and it is quite a new and extremely important aspect that might be considered. The lack of a proper attention can provoke many results e.g., serious injuries and fatal accidents. The main causes of these accidents are: stress, drugs, fatigue, high workload and emotional problems [24].

Therefore, this work presents a practical contribution regarding the *on flight* phase, including data acquisition, processing, storage and emotion recognition, analyzing it in offline mode, i.e., non real-time recognition.

## 2. Proposed Multimodal Sensing System

The multimodal sensing approach it is not a new architecture or new method to aim for a recognition system, but it is a more robust and powerful approach to be applied in situations in which a low amount of inputs (or channels) are not sufficient to reach a good recognition accuracy along the time. This approach is based on several channels (inputs) that come mainly from different sources of data. It is sometimes challenging for researchers due the time and multi sampling rate synchronization.

For some research based on contexts like emotion recognition based on physiological data, it is not recommended to use only one data e.g., heart rate variability, to accurately detect emotions, because it can reflect emotions only in strong or intense emotional situations [25]. According to some studies, when an extended number of physiological data are considered, better results can be reached.

### 2.1. Flight Experiment

A total of 13 simulated flights ($N_{flights}=13$) and eight beginner users on flight simulator ($N_{pilots}=8$) were considered, using the *Microsoft Flight Simulator Steam Edition* (FSX-SE). These flights were labeled as: RC1, RC2, RC3, GC1, GC3, LS1, LS2, VC1, VC2, CR1, CR3, CLX and CL3.

The proposed experiment corresponds to the human behaviour study of the users (pilot in command) along some proposed simulated flight tasks such as: Take off (Task 1), climbing (Task 2), route flight (cruise navigation) (Task 3), descend (Task 4), approach (Task 5), final approach (Task 6) and landing (Task 7). The environment’s setup from the main experiment, was the result of two initial Proof of Concepts (PoCs). Several improvements from these PoCs are: a large screen to improve the immersive experience during the simulation; addition of a separated computer to run the flight simulator and record facial emotions; the user must only use the joystick during the experimental flight and must use only one hand to control the flight; the GSR sensors were placed on the free hand, i.e., without movements to avoid motion artefacts; a microcontroller was used to acquire the HR data from HR device (e.g., Arduino board); the supervisor used two softwares, one to receive HR and GSR data from Bluetooth communication, and another to receive the Bluetooth data from EEG device; also a video camera was used to record the users’ body gestures.

The users were trained before the experiment regarding the flight tasks and procedures. During the main experiment, they had no contact with the experiment supervisor, who only interfered before and after each simulation. It was also recommended to the users to avoid to talk and to move the hand with GSR electrodes.

**Figure 1:**
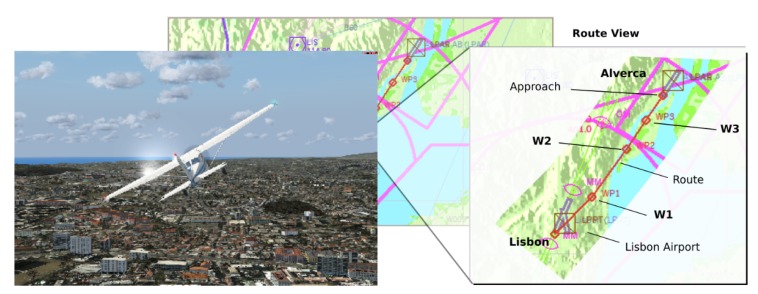
Airplane Cessna 172SP (**left**); flight route (red line) of the experiment (**right**).

All main experiments and training were executed on Visual Meteorological Condition (VMC) and minimum navigation altitude of 1800ft (feet MSL). For each user, a maximum of three flights were executed. The used airplane for this main experiment was the default aircraft model *Cessna 172SP Skyhawk*. Furthermore, the route used in this experiment, was executed with the airplane Cessna 172SP and it have almost 8.4 nm (Nautical Miles) of distance from Lisbon International Airport (ICAO LPPT/374ft/THD ELEV 378ft MSL) to Alverca (ICAO LPAR/11ft/THD ELEV 15ft MSL), as shown in Figure 1.

### 2.2. User Profile

The experiment considered users (not real pilots) of both genders between 21 and 40 years old. Considering the 13 valid flights, nine were executed by male users and four were executed by female users.

Regarding the user experience in experimental context, one male user reported to have a more deep experience in flight simulation; the other male users reported to have more experience with electronic games and all female users reported to have low experience in flight simulators and electronic games.

### 2.3. Acquisition Devices

The multimodal data acquisition was based on Heart Rate (HR), Galvanic Skin Response (GSR) and electroencephalography (EEG). The emotion monitoring system includes a set of smart sensors such as: two shimmer3-GSR+, one Medlab-Pearl100 and one Enobio-N8.

The two Shimmer3-GSR+ units were the devices used to, acquire the GSR data and to act as an auxiliary head shaking indicator, using its embedded accelerometer. It includes: 1 channel GSR (Analog); the measurement range: 10 k and 4.7 MΩ (0.2–100 µS); frequency range: DC-15.9 Hz; input protection RF/EMI filtering, current limiting; auxiliary input: 2 channel analog/I2C; digital input: via 3.5 mm; 24 MHz MSP430 CPU with a precision clock subsystem; 10 DoF inertial sensing via accelerometer integrated, gyroscope, magnetometer and altimeter; low power consumption, light weight and small form factor; also perform the analog to digital conversion and readily connects via Bluetooth or local storage via micro SD card. Furthermore, it is also a highly configurable which can be used in a variety of data capture scenarios [26].

The HR data was acquired by the Medlab-Pearl100 device. It is considered an excellent artefact0suppression device due to PEARL-technology and includes: a compact, portable and attractive design; crisp, easily readable TFT colour display; reliably measures SpO2; pulse rate, and pulse strength; integrated 100 h trend memory; integrated context sensitive help system; intuitive, multi-language user interface; works on mains and from integrated battery; full alarm system with adjustable alarm limits; usable from neonates to adults [27].

To acquire the EEG data, the Enobio Toolkit was used. It is a wearable toolkit with a wireless electrophysiology sensor system for the recording of EEG. Using the Neuroelectrics headcap toolkit (having several dry and wet electrodes), the Enobio-N8 is ideal for out-of-the-lab applications. It comes integrated with an intuitive, powerful user interface for easy configuration, recording and visualization of 24 bit EEG data at 500 sampling rate, including spectrogram and 3D visualization in real time of spectral features. It is ready for research or clinical use. In addition to EEG, triaxial accelerometer data is automatically collected. You can also use a microSD card to save data offline in Holter mode; and as like as Shimmer device, it can use Bluetooth to transmit real time data too [28].

### 2.4. Facial Emotion Sensing

During the experiment, the users’ faces and the flights along the experiments were recorded and outputs were processed after the experiment. To do this, two softwares were used: the *OBS Studio*, to record the flight and face at same time in a synchronized manner; and the software *Face Reader v.7*, a software marketed by Noldus (www.noldus.com) used to recognize the emotions based on the face recording. The last one considers seven emotions: neutral, happy, sad, angry, surprised, scared and disgust.

In offline analysis and processing, the emotions neutral and disgust were omitted. In these experiments, the Face Reader output a neutral emotion as a main emotion along each flight, which seems unrealistic because it almost omitted the amplitudes of another relevant emotions. It was confirmed by the users; they noted to not feel neutral most of the time. For this reason, we decided to omit the neutral emotion in this work. Regarding the disgust emotion, it was also omitted due to not being directly related to the flight context as confirmed by the users who they said that did not feel disgust along the flights.

**Figure 2:**
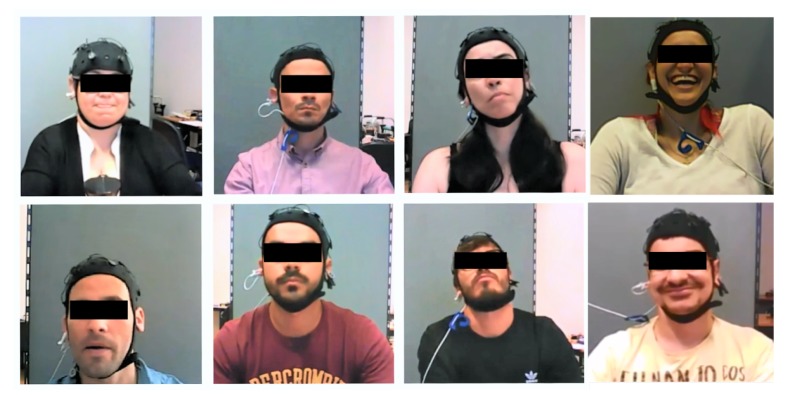
Face recording of some users during experiment.

Some users’ faces captured during the main experiment are shown in Figure 2, and it is possible to see some different reactions along the simulated flights.

The efficiency of the Face Reader software is shown in several researches and publications, being used as a reference regarding to emotion detection from facial expressions on several contexts and applications [29,30,31].

### 2.5. Physiological Sensing

The proposed multimodal sensing system considered three methods: Heart Rate (HR), Galvanic Skin Response (GSR) and Electroencephalography (EEG). To acquire these, 11 Ag/AgCl dry electrodes and one earclip were used: eight electrodes placed on the scalp (EEG), one placed on the earlobe (EEG reference), one placed on earlobe (HR) and two on the hand of the user (GSR).

The GSR data is based on Electrodermal Activity (EDA) and refers to the electrical resistance between two sensors when a very weak current occurs passed between them. It is typically acquired from the hands or fingers [6]. In this work, it was acquired by the Shimmer3-GSR+ unit, which can measure activity, emotional engagement and psychological arousal in lab scenarios and in remote capture scenarios that are set outside of the lab. It was recommended that these electrodes be kept immobile during the experiment to avoid an additional motion artifacts in GSR data.

Regarding EEG, some studies showed that it is very difficult to find the specific region on the scalp where the brain activity is sufficiently high to detect emotional states [32,33]. According to several studies, the prefrontal cortex or frontal lobe (located near the front of the head) is more involved with cognition and in decision making from emotional responses [34,35]. The 10–20 system or International 10–20 system was the method used to describe and apply the location of scalp electrodes. This way, to better detect emotion artifacts from the scalp, the electrodes were placed on that recommended area: Fp1 (channel 1), F3 (channel 2), C3 (channel 3), T7 (channel 4), Fp2 (channel 5), F4 (channel 6), C4 (channel 7) and T8 (channel 8). The EEG reference electrode (EEGR) was placed on the user’s earlobe. It frequency aimed our choice to use the beta rhythms (or band) in this experiment [32,36].

**Figure 3:**
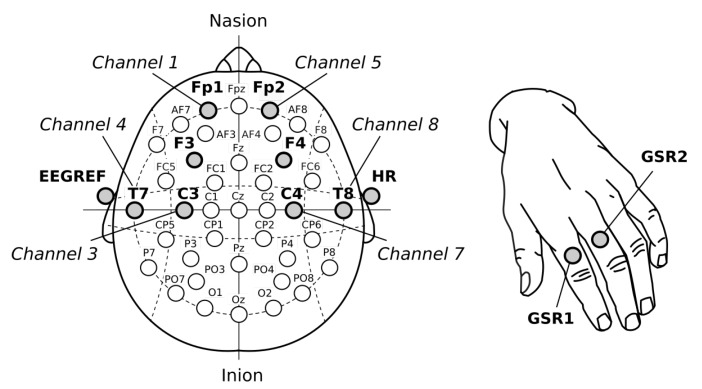
Electrodes placement. EEG and HR, placed on the scalp and earlobe (**left**); and GSR, placed on the indicator and middle fingers (**right**).

Figure 3, shows the electrodes position used during experiment. Note the use of the frontal cortex to acquire EEG data, which the beta rhythm (β-band) were considered i.e., brain signals between 12 and 30 Hz.

**Figure 4:**
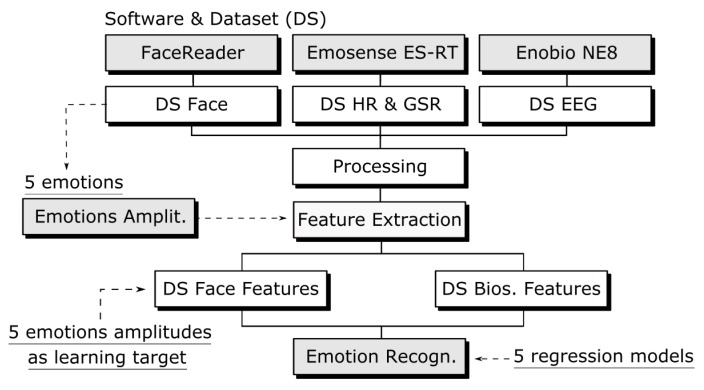
Datasets used in this work.

Putting all datasets together, it is possible to see the role of each one in this work (Figure 4). One dataset is produced by the Face Reader v7.0 and it outputs in real time the amplitudes of five emotions along the time.

This research does not process face expression to detect emotions, instead, the Face Reader does it for us and outputs five emotional amplitudes which are used to lead the emotion recognition task during the Deep Learning and ANN training over another dataset based on biosignals, both synchronized in time.

## 3. Feature Extraction

Feature extraction is the last step before data recognition or classification. It is very important in pattern identification, classification, modeling and general automatic recognition. Its importance is fundamental to minimize the loss of important information embedded in the data [37] and to also optimize a dataset, giving clearer information to recognize any pattern.

This work uses different feature extraction techniques according to the technique used to acquire the physiological data (e.g., HR, GSR and EEG). Its extraction was executed after the processing phase which prepared the data to have more clear features. It was applied over time and frequency contexts are better described in the next section.

### 3.1. Features Description

In this work, we extracted 15 different features based on time and frequency. Each feature was chosen according to each dataset characteristics as presented in Table 1, which describes all extracted features, as such as the correspondent datasets. If the dataset needed a frequency analysis, it was applied through its features such as EEG datasets which used filtering and other analysis in frequency and time.

Regarding to GSR datasets, it was important to understand its data profile and behaviour to properly relate it to the amount of peaks (peaks frequency) along the time/events; for this reason, one feature that relates peaks by time, was applied. Other peculiarities are also found over the HR datasets as, for instance, the HR variabilities during several emotional events along time. This HR dynamic fluctuation along the time, were mainly represented by three features. Furthermore, several statistical features were also applied over all datasets, along time, considering several sample lengths.

### 3.2. Wavelets (FEAT_WAC, FEAT_WDC)

The wavelet analysis plays an important role as part of the feature extraction methods. It allows to analyze time and frequency contents of data simultaneously and with high data resolution. When applied over a continuous data, it is called Continuous Wavelet Transform (CWT), and over a discrete data, it is Discrete Wavelet Transform (DWT) [38] (Equation (1)).

(1) $$CWT\left(a,b\right)=\int_{-\infty }^{+\infty }x\left(t\right)\psi_{a,b}^{*}\left(t\right)dt,$$

where *x*(*t*) represents the unprocessed data, *a* is the dilation, and *b* is the translation factor.

It lies on the concept of *mother wavelet* (MWT), which is a function used to decompose and describe the analyzed data. The Symlets (‘sym7’) was the MWT used, due its high similarities and compatibilities with the EEG data on all scalp regions [39].

Furthermore, as shown previously, the CWT method includes a complex conjugate term denoted by $\psi_{a,b}^{*}$, where ψ(t) means wavelet [37] (Equation (2)).

(2) $$\psi_{a,b}\left(t\right)=\frac{1}{\sqrt{\left|a\right|}}\psi \left(\frac{t-b}{a}\right).$$

### 3.3. Continuous Entropy (FEAT_ENT)

The continuous entropy or differential entropy is another feature used in this work. It is a concept in data theory to represent the measurement of the average rate of a random variable; it is also understood as a method to measure the quality or class diversity of such datasets. On *continuous probability distributions*, it is based on the expansion from *Shannon entropy* concept, defined by Equation (3),

(3) $$h\left(X\right)=-\int_{0}^{N(S)}f\left(x\right)logf\left(x\right)dx.$$

where *X* represents a random variable defined by a probability density function of a subset *S*.

### 3.4. Sample Absolute Interval Range (FEAT_RNG)

The range of a sample was also used as a feature. It is defined as the absolute difference between the values compared to the last f(t) and the first position f(t−Δt) of a sample in time, as shown in Equation (4), which Δt represents the interval length to displace the interval from the actual position *t*.

(4) R(t)=|f(t)−f(t−Δt)|

### 3.5. Poincaré Plots (FEAT_SD1, FEAT_SD2, FEAT_SCT, FEAT_SAR)

The Poincaré plots of RR intervals is one of the methods used in Heart Rate Variability (HRV) analysis. It returns a useful visual map (or cloud), which is capable to summarize the dynamics of an entire RR time series regarding to actual and next one values. It is also a quantitative method to give information over the long- and short-term HRV [40,41].

**Figure 5:**
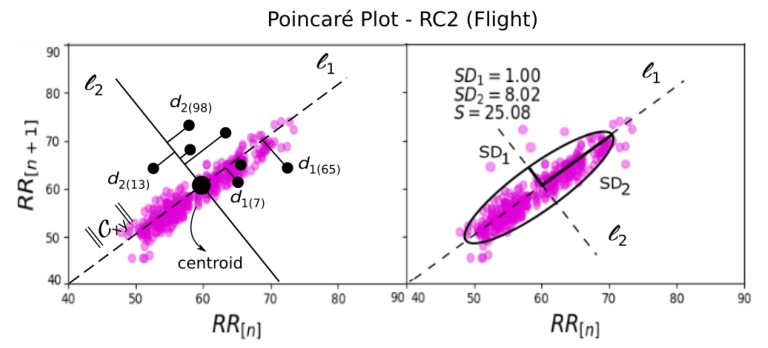
Poincaré plot demonstration over the flight dataset RC2.

This method is represented by Poincaré *descriptors*, SD1 and SD2, which are used to quantify geometrically the produced cloud. It is given in terms of the variance of each $RR_{i}$ and $RR_{i+1}$ pairs. The *i* refers to the *i*th RR value, as shown in Figure 5.

Mathematically, let the HRV be defined by the vector $RR=[RR_{1},RR_{2},\ldots ,RR_{n+1}]$ and the position-correlated vectors *x* and *y* defined as [41,42],

(5) $$x=\left[x_{1},x_{2},\ldots ,x_{n}\right]\equiv \left[RR_{1},RR_{2},\ldots ,RR_{n}\right],$$

(6) $$y=\left[y_{2},x_{3},\ldots ,y_{n+1}\right]\equiv \left[RR_{2},RR_{3},\ldots ,RR_{n+1}\right].$$

For a regular Poincaré plot, the centroid vector $C_{xy}=\left[x_{c},y_{c}\right]$ of its cloud representation, is define by,

(7) $$x_{c}=\frac{1}{n}\sum_{i=1}^{n}x_{i},y_{c}=\frac{1}{n}\sum_{i=1}^{n}y_{i}.$$

To compute the numerical representation of the centroid, the *vector norm* is applied using the Equation (8).

(8) $$\left|\right|C_{xy}\left|\right|=\sqrt{x_{c}^{2}+y_{c}^{2}}$$

To compute the *descriptors* (short-term variability) SD1 and SD2 of a standard Poincaré plot, the distances $d_{1}$ and $d_{2}$ of any *i*th RR from the centroid *interceptors *$l_{1}$ and $l_{2}$ respectively are defined as,

(9) $$d_{1i}=\frac{|\left(x_{i}-x_{c}\right)-\left(y_{i}-y_{c}\right)|}{\sqrt{2}},d_{2i}=\frac{|\left(x_{i}-x_{c}\right)+\left(y_{i}-y_{c}\right)|}{\sqrt{2}}$$

Considering those prior algebraic definitions for a *standard cloud*, it is possible to compute the SD1 and SD2.

(10) $$SD1_{c}=\sqrt{\frac{1}{n}\sum_{i=1}^{n}d_{1i}^{2}},SD2_{c}=\sqrt{\frac{1}{n}\sum_{i=1}^{n}d_{2i}^{2}}$$

The area covered by the resulted ellipse, was also used as a feature for HR dataset, and it can be determined as below.

(11) SA=π.SD1.SD2

### 3.6. Singular Value Decomposition: Features Selection

When the features are extracted, some of them can be useless in the recognition process; to select the best set of them (i.e., to do a *dimensionality reduction*), the Singular Value Decomposition (SVD) was used, executing a *matrix decomposition* or *matrix factorization* of the input matrix. It is based on eigenvalues, applied to a bidimensional *m×n* matrix *A*. Mathematically, this method factorizes a matrix into a product of matrices, as shown in Equation (12).

(12) $$A=UDV^{*},$$

where *D* is a non negative diagonal matrix having the singular values of *A*; *U* and *V* are matrices that satisfy the condition $U^{*}U=I$, $V^{*}V=I$. The resultant matrix of that decomposition is the new input applied into the recognition process.

## 4. Emotion Recognition

The emotion recognition uses Artificial Neural Networks (ANN) and Deep Learning techniques (DL). The Multilayer Perceptron (MLP-ANN) architecture, Back-Propagation and Deep Learning algorithms were developed over the *Python3* Toolkits, *PyBrain*, *Keras* and *TensorFlow* having execution support of the Graphics Processing Unit (GPU).

The ANN is a supervised technique, inspired by human brain behaviour; it can process several instructions in short periods of time, taking fast decisions and reactions. Its architecture can be designed according to the problem to be solved. A small number of neurons is recommended for simpler problems. If the problem complexity increases, a new amount of neurons must to be analysed as needed. Each single neuron represents a single function over several parameters of activation and thresholds/biases. The techniques based on neural networks e.g., ANN, CNN, RNN, DNN, are powerful tools due their high capacity to solve complex tasks, being massively used in modern controls, dynamic systems, data mining, automatic bio-patterns identification (e.g., fingerprints or face recognition) and robotics. We can also cite the high capacity of the ANN to produce complex and parallel solutions over the field of features, which each ANN layer can presents different and parallel outputs to converge on final functions or probabilistic outputs. It does not mean that it cannot be applied, combined with other techniques such as, K-Means or SVM, for instance.

The final emotions are recognized from the biosignals and are based on ANN training using the labels produced by the Face Reader. In other words, initially, the system uses the emotions’ labels processed by the Face Reader, synchronizes it in time with the biosignals and uses these labels in the training phase to teach the ANN to predict or recognize new emotions using only the biosignals.

### 4.1. ANN Development and Modeling

The training data (partial set of features) is defined in Equation (13), where τ represents the training-set, x(n) the input-set (or input data features), d(n) the desired output in each iteration *n* (due the use of a supervised learning method), and $N_{i}$ that represents the amount of instances from the training-set [43].

(13) $$\tau =\left\{x\left(n\right),d\left(n\right)\right\}{|}_{n=1}^{N_{i}}$$

The *Induced local field* (for forward computation) was used and can be computed by Equation (14), which $x_{i}$ goes from input neurons *i*, $w_{ji}$ and $w_{b}$ represent the weights connections from the neuron *j* to *i*, and $b_{ji}$ is the bias applied for each neuron, by iteration *n*.

(14) $$v_{j}\left(n\right)=\sum_{i=1}^{N}w_{ji}\left(n\right)x_{i}\left(n\right)+b_{ji}w_{b},j\geq 1$$

For each hidden layer, two different activation functions were considered: the sigmoidal and ReLU. The *sigmoidal activation function* φ(·) is defined by Equation (15), where *a* determines the threshold’s function. The sigmoid function returns values between 0 and 1.

(15) $$\varphi_{j}{\left(v_{j}\left(n\right)\right)}_{sig}=\frac{1}{1+e^{-av_{j}\left(n\right)}},a\geq 1$$

Another activation function applied in this work, is the *ReLU* or *rectified linear unit*. It is defined by Equation (16), which returns values between 0 and +∞.

(16) $$\varphi_{j}{\left(v_{j}\left(n\right)\right)}_{ReLU}=\left\{\begin{array}{cc} 0 & \mathrm{if}\;x<0\\ x & \mathrm{if}\;x\geq 0 \end{array}\right.\;$$

Regarding to the output layer, also two different activation were considered. The prior active functions and the *softmax activation function* P(y|X), which it is applied as defined by Equation (17). It represents the prediction probability for each emotion in all $N_{o}$ output neurons.

(17) $$P\left(y=j|X\right)\left(n\right)=\frac{e^{v_{j}\left(n\right)}}{\sum_{k=1}^{N_{o}}e^{v_{k}\left(n\right)}}$$

the P(y|X) is mainly applied, when we are facing a classification problem i.e., which the outputs return independent probabilities for each considered class in case. Otherwise, when using the $\varphi_{j}\left(v_{j}\left(n\right)\right)$, the ANN output can be represented by any amount of neurons, which it must return independent values (not probabilities), being useful when we are working with regression analysis. Since this work lies over the ANN and regression problems, the $\varphi_{j}\left(v_{j}\left(n\right)\right)$ was used.

The error data or *instantaneous error* produced by output layer of each neuron *j*, is defined by Equation (18),

(18) $$\varepsilon_{j}\left(n\right)=d_{j}\left(n\right)-y_{k}\left(n\right)$$

where $d_{j}\left(n\right)$ represents the *j*th element of d(n) and $y_{k}\left(n\right)$ the *k*th *instantaneous output*. Furthermore, the $y_{k}\left(n\right)$ and the *instantaneous error energy* (ξ) of each neuron *j* (Equation (19)) are both considered to reach best network accuracy along epochs (iterations) [43,44].

(19) $$\xi_{j}\left(n\right)=\frac{1}{2}\varepsilon_{j}^{2}\left(n\right)$$

The local *gradient* applied to each neuron *k* from the output layer, is described by Equation (20).

(20) $$\delta_{k}\left(n\right)=\varepsilon_{k}\left(n\right)y_{k}\left(n\right)\left(1-y_{k}\left(n\right)\right)$$

The general ANN weights adjustments (for backward computation) applied to each output neuron, is defined by *delta-rule* [43] (Equation (21)),

(21) $$\Delta w_{kj}\left(n\right)=\alpha \Delta w_{kj}\left(n-1\right)+\eta \delta_{k}\left(n\right)y_{k}\left(n\right)$$

where the *momentum* α ([0; 1]) is used to avoid learning instabilities while increasing the *learning rate* η ([0; 1]), decreasing the mean error; furthermore, both are adjusted during the training phase.

### 4.2. Cross Validation—Testing Recognition Models

All the emotion recognition test were executed based on the methodology of Leave-One-Out Cross Validation (LOOCV).

The LOOCV was shown to be a good methodology on the proposed multimodal system to support the emotions recognition from each pilot, based on the learned emotions captured from the prior flights. It leaves one flight dataset out, while it trains the ANN using the other flight datasets.

### 4.3. Realtime Outliers Removal—RTOR

**Figure 6:**
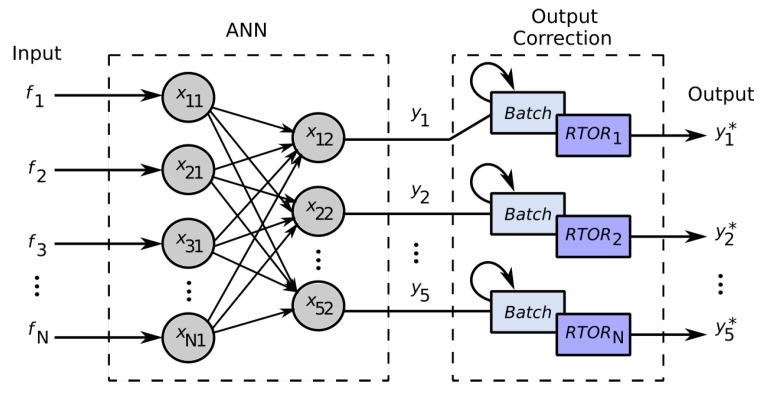
RTOR operation diagram.

Sometimes, the neurons output abrupt values; wrong values are critical to compute the evaluation metrics (e.g., the absolute mean errors) correctly. To correct these realtime abrupt outputs, the Realtime Outliers Removal (RTOR) method was developed in this work. It is based on the last *N* output samples (based on a *batch* to store realtime samples acquisition) to eliminate the actual outlier from each output at same time (Figure 6).

**Figure 7:**
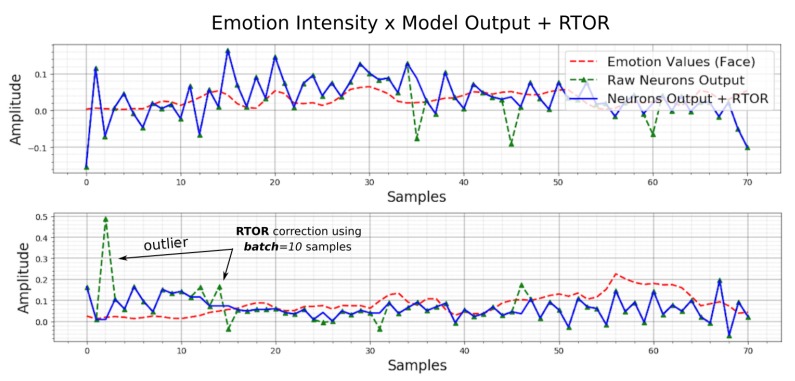
Realtime outlier correction based on RTOR method. Note the corrected output (blue) and the raw output with outliers (green).

Figure 7, shows in practice, the correction of two neuron outputs (top and bottom plot), representing two intensities of emotion outputs. The dotted red line, represents the target (emotion detected from face); the blue line, represents the corrected output (RTOR); and the dotted green line, represents the raw output. The relative output outliers detection and removal, are controlled by a batch length, which represents the amount of samples to be treated in realtime.

### 4.4. Evaluation Metrics for Emotion Output: Regression Models

Before the metrics presentation to evaluate the emotion recognition outputs, it is extremely important to know that this work does not consider one single emotion as the final output, but the intensities of several emotions i.e., five emotions by time, output by each independent output neuron. This is because the most human bodies do not usually feel one single emotion at a time, but several of them, having different intensities and valences. For this reason, the presented evaluation metrics work over regression outputs over all output neurons measured separately.

Each output neuron was designed as a regression function and trained to output emotion intensities using the emotion detected from the face as the target. These outputs are measured to analyze how close the outputs are from the targets.

#### 4.4.1. Root Mean Squared Error (RMSE)

Considering the prior $R^{2}$, the Root Mean Squared Error (RMSE) or also called, Root Mean Squared Deviation (RMSD), computes the error distance between the estimated values $\hat{y}\left(n\right)$, as defined below.

(22) $$RMSE=\sqrt{\frac{\sum_{n=1}^{N}{\left(\hat{y}\left(n\right)-y\left(n\right)\right)}^{2}}{N}}$$

#### 4.4.2. Mean Absolute Error (MAE)

The MAE represents the average of the absolute difference between the predicted values and observed value (prediction). In another words, it is a linear representation, which means that all the single differences are weighted equally in the average as shown in Equation (23):

(23) $$MAE=\frac{1}{N}\sum_{n=1}^{N}\left|y\left(n\right)-\hat{y}\left(n\right)\right|$$

## 5. Result Analysis

This work presented a multimodal solution to recognize emotions from several physiological inputs based on the bio-reactions from beginner users of flight simulator. It is an important contribution regarding aviation and a more general perspective of emotion relationship. It was proposed as one way to contribute to emotion studies and in this work, the context of application was mainly the aviation side of the scope of aviation accidents, which were caused by human failures.

Several tests were executed in this work to try to find recognition results for each pilot, i.e., the best model possible to try to estimate emotions felt by each pilot during the flight experiment. The cross-validation was the method used to aim the emotions recognition process for each pilot dataset obtained during each flight experiment. The recognition tasks were initially based on two different tests: *tests without feature extraction* (i.e., raw data directly applied in ANN inputs, with any treatment or preprocessing) and *processed data with feature extraction*. Were also considered different ANN architectures, amount of training iteration, amount of inputs and hidden neurons, and different flight datasets.

In all emotion recognition tests, the cross validation was applied to recognize the emotions felt by the pilot in a single flight according to the emotions already detected from another flights. In other words, the training was based on 12 flight datasets (N−1 flights), to try to recognize the emotions from one single flight. It is important considers, that the dataset having emotions values from the face (5 different emotions), was the reference of the ANN training. For this reason several mistakes from the facial reader software, detecting wrongly several emotions, were not possible to be avoided; the consequence of these wrong matches is several errors under the regression models, outputted from each output neuron.

### 5.1. Description of the Recognition Tests

The main procedures applied from the processsing and feature extraction, are shown in the test sequence below. It was based on feature selection and data type of treatment. For most of the tests, at least the data normalization and abrupt data correction were used (Table 2). In these tests, we considered all the features from each data, i.e., 11 features from HR, seven features from GSR and 72 features from EEG (9 × 8 Ch), including the best and worst features.

Between tests 19 and 34, we considered the features selection based on SVD (it means that now, the features are selected in order of importance). There were six features from HR, four features from GSR and 40 (5× 8 Ch) features from EEG, as presented in Table 3.

### 5.2. Emotion Recognition Tests Based on Raw Data: Test 1 and Test 2

In these tests of emotion recognition, no feature extractions and preprocessing were considered; all raw data were directly applied in the ANN input layer. The ANN activation function was sigmoid and two different optimization algorithms: stochastic gradient descend (‘sgd’) and ‘adam’.

Table 4, presents an emotion recognition result using a raw data approach and no feature extraction. Its results show the importance of feature extraction in a multimodal sensing system in which without it, the recognition will have more undesirable results and high execution time. The RMSE and MAE were used to compare the output models with the emotions from the flight datasets.

### 5.3. Emotion Recognition Tests Based on Feature Extraction: Test 3 to 34

All tests between 3 and 34 considered feature extraction over the raw input data. In detail, between tests 3 and 18, 90 features were extracted, considering all features. Between tests 19 and 34, the SVD was applied to select the best features to be used. Table 5 presents the tests results, referring to tests 11 and 12, based on feature extraction and, in this case, without features selection.

The accuracy of the *match* procedure i.e., the correct match in each sample regarding to the higher emotion amplitude (between five emotions), presented the worst values on recognition from the flight dataset CLX, having any match on most recognition, surely due its high noises and small number of samples analysed before and after the feature extraction, which changed from 518 to 10 samples.

### 5.4. Emotion Recognition Analysis

**Figure 8:**
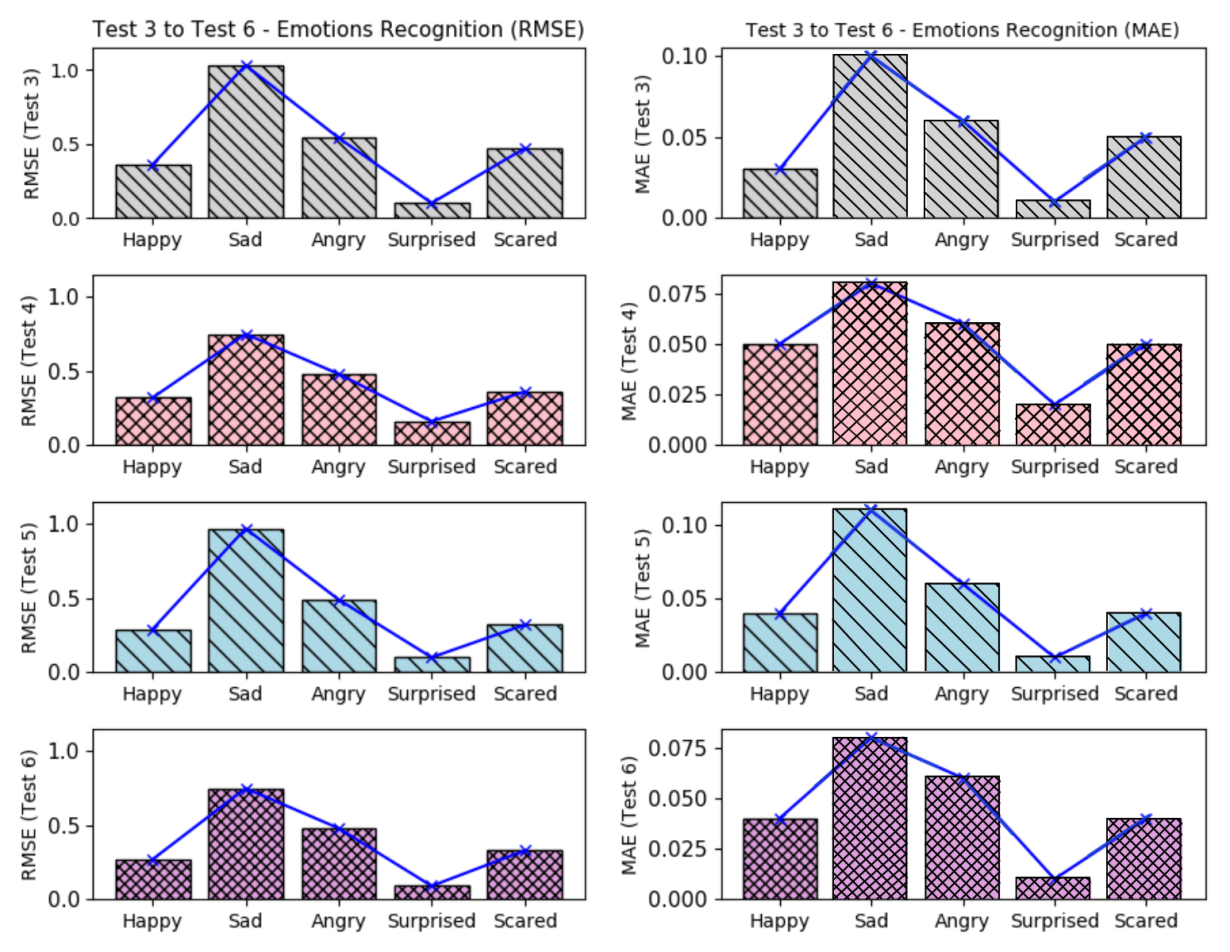
Errors results (RMSE+MAE) from tests 3–6 (with feature extraction).

Figure 8, presents the barplots correspondent to the errors results from the tests 3–6, with feature extraction but without feature selection and considering all three data.

**Figure 9:**
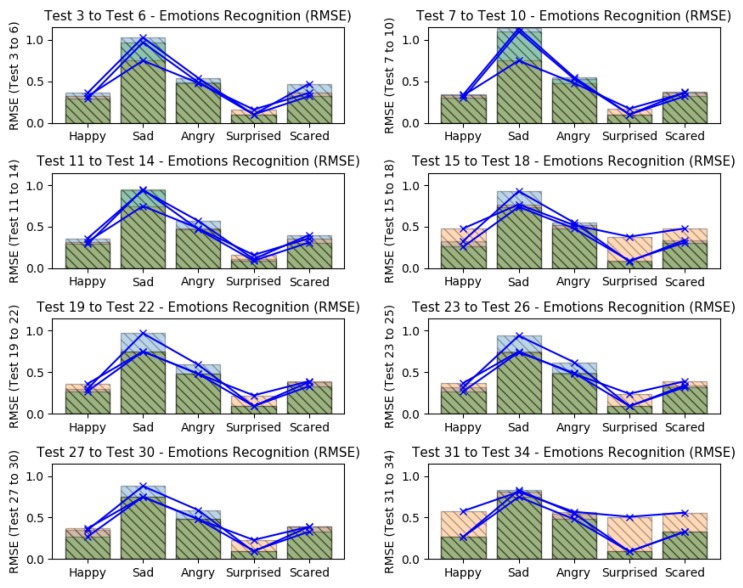
Errors results (RMSE) comparison from tests 3–34 (with feature extraction).

**Figure 10:**
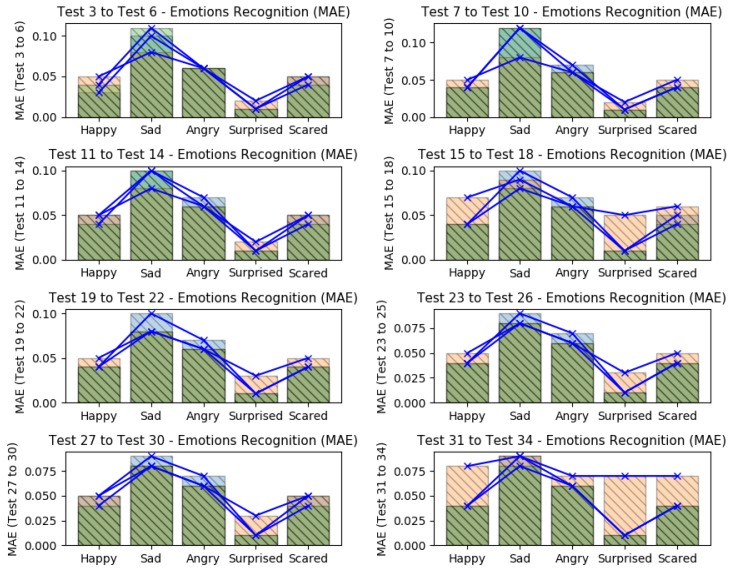
Errors results (MAE) comparison from tests 3–34 (with feature extraction).

It is possible to see that in tests 3–6, the emotion *surprised* presented a higher recognition accuracy, having the smallest error level. The *happy* and *scared* were the emotions which also presented low errors. Nevertheless, these error levels can be improved if the training datasets are more coherent. The emotions *sad* and *angry*, presented the worst error levels; it is probably due the misclassifications from the face emotiom detection software, which sometimes confused situations of angry and disappointed rather than sadness. If we compare all tests (from test 3–34), it is possible to note that again, the *surprised* emotion kept with best recognition values (low errors), as shown in Figure 9 and Figure 10, which it present all considered errors along the tests.

The higher recognition errors were reached when the EEG datasets were omitted in different tests (tests 15–18 and tests 31–34), showing that in these tests, the recognition results were better when all data were considered; when GSR datasets were ommited, the results got good predictions too (tests 11–14 and tests 27–30). The application of *feature selection* based on SVD and the omission of GSR datasets, returned the less recogmition errors (tests 27–30). The *sad* emotion got the worst error levels when HR datasets were omitted (tests 7–10), as like as the *happy* emotion got the worst error levels when the EEG datasets were omitted.

In summary, all tests showed that the smallest error levels can be reached when all datasets were considered or when the GSR datasets were omitted. Also, they showed that the emotion *surprised* was easier to expect, having a mean RMSE of 0.13 and mean MAE of 0.01; while the worst predictions were found to emotion *sad*, having a mean RMSE of 0.82 and mean MAE of 0.08.

### 5.5. Improvements Coming from the Feature Extraction

**Figure 11:**
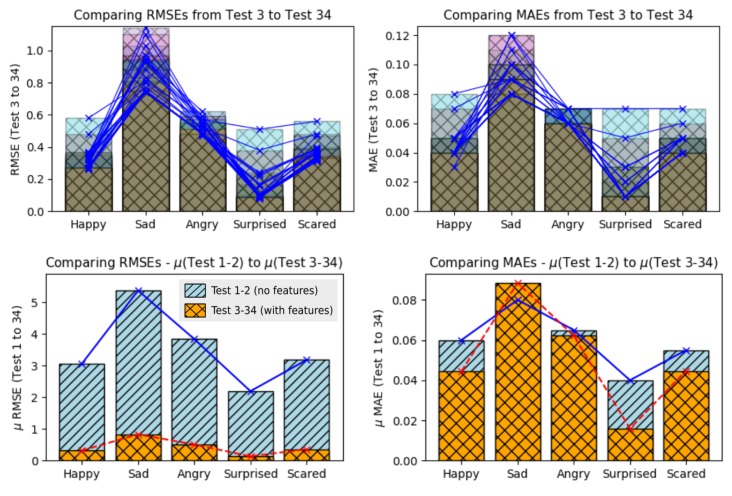
Errors results comparison between RMSE and MAE from tests 1 to 34 (with feature extraction).

In prior discussion, we presented the need to use features extraction in a very dense datasets. One direct benefit of it is the execution time. With the feature extraction, the dataset is sampled to fractions of data which it must continue to represent all raw data with more or equal meaning. For this reason, a featured dataset is smaller if compared to its raw dataset. Another benefit of feature extraction is that it can brings information from a dataset in statistical or frenquency context, e.g., data variances and other tiny patterns of the frenquency domain. Figure 11 shows the errors levels between the use of *raw datasets* (tests 1 and 2) and *featured datasets* (tests 3 to 34).

Analyzing the RMSE values (left barplot), it is possible to see that the improvements were important over all emotions when feature extraction was used. The emotion *happy* presented an improvement of 89.66% (prior 3.06/actual 0.31); *sad* of 84.58% (5.38/0.82); *angry* of 86.75% (3.84/0.50); *surprised* of 93.89% (2.19/0.13); and *scared* of 88.67% (3.18/0.36).

Analyzing the MAE values (right barplot), it is possible to see that the improvements were good over 4 emotions of 5 (emotion *sad* wasn’t improved on MAE values), when feature extraction was used. The emotion *happy* presented an improvement of 26.04% (prior 0.06/actual 0.04); *angry* of 4.32% (0.065/0.062); *surprised* of 60.15% (0.04/0.01); and *scared* of 18.75% (0.05/0.04).

### 5.6. Considering the Higher Intensities Between Emotions

The higher emotion intensities by time (between five emotion intensities) were also computed and its amount of matches were also analyzed, comparing the correct matches betewen its higher emotion (from the face dataset) with the higher output from the five neurons (output layer).

The benefit to also consider these higher values by time is to understand if the regression models from each output neuron is following the original emotion intensities related to the other emotions. In other words, if a regression model from each neuron, fits to a target, both *errors levels* (RMSA+MAE) and *major/higher values* will improve together.

**Figure 12:**
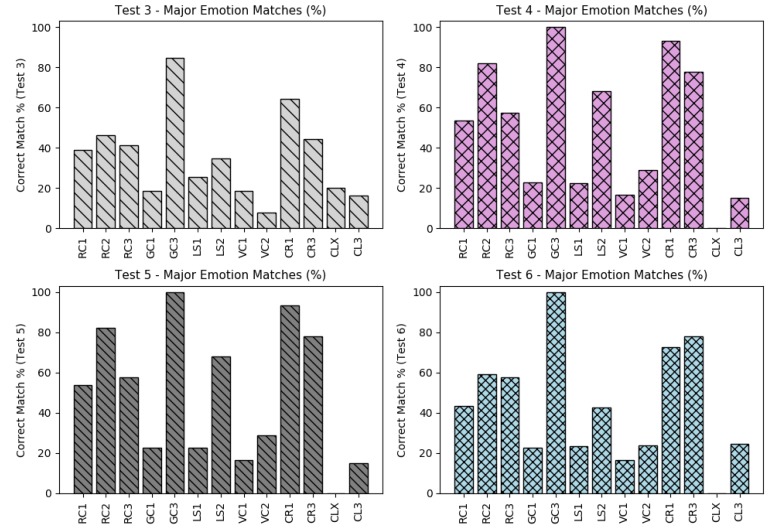
Major emotion accuracies from the tests 3 to 6 (with feature extraction).

The corrected amount of matches between these emotions and its relations is shown in Figure 12, presenting the case of tests 3–6.

Some datasets presented a very low amount of matches during all tests as for instance, *GC1*, *LS1*, *VC1*, *CLX* and *CL3*. These low accuracies are probably due the high misclassification of emotions from the pilots’ faces as also presented on prior errors values based o RMSE and MAE. However, if we consider the possibility to improve these results, the next tests can omit these datasets with low accuracies to get better general results.

When comparing all matches (from test 3–34) regarding to the major emotion values, it is possible to see that the accuracy of the dataset *CLX* continues to present the worst accuracies and the dataset *GC3* the best accuracies values.

**Figure 13:**
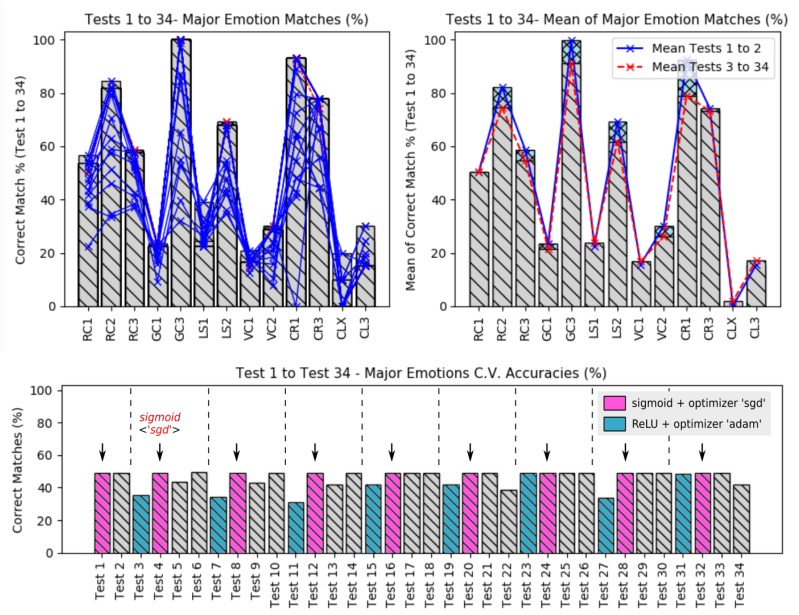
All higher emotion accuracies from tests 1–34. All accuracies (**left**); mean of all accuracies (**right**).

Figure 13, shows a comparison of all accuracies, regarding to the major emotions from the tests 3 to 34 (top plots) and from tests 1 to 34 (bottom plot). Note that on the top plot, shows that six datasets kept the major emotion accuracies less than 50%.

The top left plot, presents the relation between the mean of the raw dataset accuracies (tests 1 and 2) over the featured datasets accuracies (tests 3–34), which the raw data tests seems to have better accuracies over the feaured dataset. It means that the recognition based on raw datasets was the best solution in this proposed work.

The answer for that is *not necessarily*; if we go back a little and observe the error levels during the tests based on raw datasets, we will see that it was extremily bad compared to the others tests based on featured dataset; this way we can easily note that actually, a good regression model must be based on a combination of low error levels and good major emotion accuracies.

Finally, when analizing the bottom plot, it is possible to note that when the activation function was the *sigmoid* together with the *gradient descend* optimization, the general accuracies presented a constant behaviour along the executed tests. The activation function *rectified unit* presented the worst major emotion accuracies in this work.

### 5.7. Improving These Results

To improve these results, this work shows that is strongly recommended, to first, to optimize the emotions detection from the face. It were undoubtedly, the main reason for several undesirable recognition error levels. Another way to improve it is to omit some datasets which presented bad predictions; it surely will improve the general predictions or emotional recognition.

However, some results were already improved during this work. For instance, when looking to the learning tasks, absolute improvements, were applied, changing the *traditional learning* techniques by the *Deep Learning* techniques. These last improvements optimized the resognition results in *accuracies* of recognition and in *execution time*.

**Figure 14:**
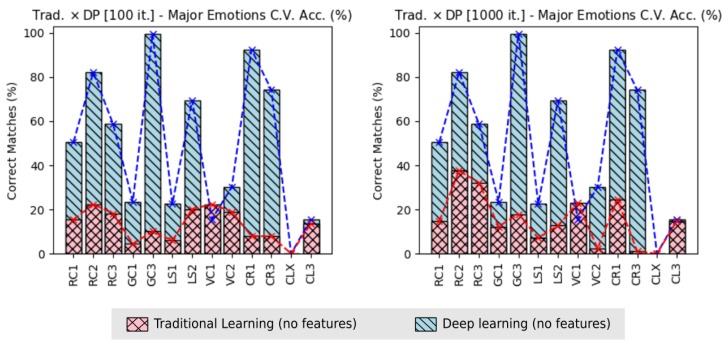
Traditional learning versus Deep Learning (DP). Improvement applied in this work regarding to the major value emotions when applying the traditional learning and Deep Learning (no feature extraction).

Figure 14, shows the improvement due the use of *Deep Learning* techniques, regarding to the amount of correct matches of the major emotions values, between all emotions considered in this work. It is possible to see, that the dataset CLX kept with worst accuracy also on traditional learning.

Regarding the accuracies of the major value emotions based on 100 training iteration of the traditional learning, the improvement happened in 11 flight datasets from 13: RC1 was improved in 69.52% (prior 15.39/actual 50.50); RC2 72.71% (22.41/82.13); RC3 of 68.97% (18.25/58.83); GC1 of 80.97% (4.48/23.55); GC3 of 89.88% (10.08/99.65); LS1 of 73.63% (5.93/22.49); LS2 of 70.96% (20.16/69.43); VC2 of 37.08% (18.95/30.12); CR1 of 91.40% (7.95/92.47); CR3 of 89.39% (7.87/74.18); and CL3 of 12.13% (13.68/15.57). The higher and lower improvements happened for datasets CR1 and CL3, respectively.

Considering the traditional learning using 1000 training iteration, the improvement happened in 11 flight datasets from 13, as in prior situation: RC1 was improved in 70.77% (14.76/50.50); RC2 of 54.25% (37.57/82.13); RC3 of 45.31% (32.17/58.83); GC1 of 47.77% (12.30/23.55); GC3 of 82.00% (17.93/99.65); LS1 of 68.25% (7.14/22.49); LS2 of 81.17% (12.69/69.43); VC2 of 92.19% (2.35/30.12); CR1 of 73.36% (24.63/92.47); CR3 of 98.53% (1.09/74.18); and CL3 of 5.20% (14.76/15.57). The higher and lower improvements happened for dataset CR3 and CL3 respectively.

The improvements of accuracies over the major emotion values at 100 training iterations were higher, because the execution with 1000 training iterations presented better accuracies (i.e., less difference from Deep Learning); however, due the very high exponential execution time of the tradition learning, it demotivate the execution of it traditional manner, using the same training iteration used with the Deep Learning (6000 training iterations), which can take days or weeks.

If we consider the improvements over the *execution time*, the use of Deep Learning instead the traditional methods, we notice an optimization of 92.17%, having 4406.32 s (mean of the Deep Learning applied on tests 1 and 2) instead of 56,321.40 s (traditional learning), even when the amount of training iteration was 60 times less, i.e., 100 over 6000 the from Deep Learning. When the training interation of the traditional learning was increased to 1000, the improvement with the use of Deep Learning was 99.09%, having 4406.32 s (Deep Learning) instead of 484,586.47 s from traditional learning, even using 6 times less training iterations.

Another way to improve the final results, is to exeute more flight tests, increasing the amount of data in the dataset. Also, applying personal dataset concept, which the emotion recognition should also be based on personal characteristics of each pilot.

### 5.8. Emotions Instances from Face Expressions

**Figure 15:**
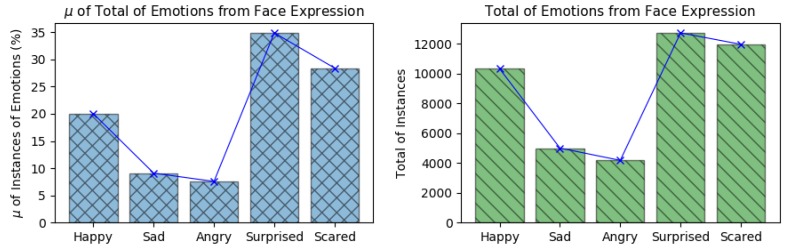
Amount of emotions instances detected by the Face Reader v7.0.

The emotion amplitudes detected by the Face Reader v7.0 were used as the emotion references during the emotion recognition phase using all biosignals based on Deep Learning and ANN. Each emotion instance detected during all flights executed in this work is shown in Figure 15-left, which shows the mean of the percentage of emotion instances along the 13 flights and in Figure 15-right, which shows the total amount of emotion instances detected along all flights.

According to the Face Reader outputs, during the flight experiments, the pilots experienced more of: *happy*, *surprised* and *scared*. Emotions *sad* and *angry*, presented less occurrences along the experiments. These outputs are in line with the arguments presented by each pilot during the experiments.

These emotion instances presented a relation with the amount of recognition errors presented during the emotion recognition phase. It is because the emotions *happy*, *surprised* and *scared* presented more instances along the experiments (more instances to train), what it resulted on the lower errors levels along the emotion recognition.

## 6. Conclusions and Future Work

This work presented a solution to detect emotions from pilots in command (i.e., beginner users on a flight simulator), during simulated flights. These flights were executed by the Microsoft Flight Simulator Steam Edition (FSX-SE), using a Cessna 172SP aircraft. The users from the experiment were beginners in simulated flights and they were trained before. A total of seven flight tasks were defined such as: take off, climbing, navigation, descent, approach, final approach and landing.

We considered three different data from the pilots’ bodies: HR, GSR and EEG. They were acquired at the same time during the flight based on several sensors such as Enobio-NE8, Shimmer3-GSR+, MedLab-Pearl100 and Arduino.

After data acquisition, the processing was executed to correct abrupt changes of the data, to detrend, remove outliers, normalize the data and execute filterings and data sampling. The feature extraction was executed over the processed data where several features were extracted to aim for the recognition phase. The ANN was used to recognize emotions using the extracted features such as the ANN inputs, based on traditional and Deep Learning techniques.

The emotion recognition results reached different levels of accuracy. The tests of the produced output models showed that the lowest recognition errors were reached when all data were considered or when the GSR datasets were omitted from the model training. It also showed that the emotion *surprised* was the easiest to analyse, having a mean RMSE of 0.13 and mean MAE of 0.01; while the emotion *sad* was the hardest to recognize, having a mean RMSE of 0.82 and mean MAE of 0.08. When were considered only the higher emotion intensities by time, the most matches accuracies were between 55% and 100%. It can be partially explained by the amount of emotion instances detected by the Face Reader, which the emotions *happy*, *surprised* and *scared* presented more instances along the experiments.

As part of future work, we intend to execute more emotion recognition tests, omitting the datasets that presented the lowest accuracies (considering the matches with the higher emotions by time), to optimize the total mean accuracies. Also, we aim to optimize the quality of the face emotion dataset, processed by the Face Reader software, and then to obtain higher accuracies and lower error levels. Increasing the number of flight experiments is another improvement that can be applied in future work; it would generate more data for training during the recognition phase.

## Figures and Tables

**Table 1:** Features extraction for HR, GSR, EEG and Face datasets.

Ord.	Extracted Features	Feature Description	Applied to Dataset (s)
1	**FEAT_MN**	⋄ Mean of a sample.	HR, GSR, EEG, Face
2	**FEAT_MD**	⋄ Middle value of a sample.	HR, GSR, EEG, Face
3	**FEAT_STD**	⋄ Standard deviation (σ) of a sample.	HR, GSR, EEG
4	**FEAT_VAR**	⋄ Variance ($\sigma^{2}$) of a sample.	HR, GSR, EEG
5	**FEAT_ENT**	⋄ Measure the samples’ entropy i.e., irregularities.	HR, GSR, EEG
7	**FEAT_RNG**	⋄ Absolute range (*max* − *min*) value of a sample.	HR, GSR, EEG
8	**FEAT_RMS**	⋄ Root mean squared of a sample.	HR, GSR, EEG
9	**FEAT_PEK**	⋄ Measure the amount of peaks into a sample.	GSR
10	**FEAT_WAC**	⋄ Mean of the wavelet approximation coefficient.	EEG
11	**FEAT_WDC**	⋄ Mean of the wavelet detailed coefficient.	EEG
12	**FEAT_SD1**	⋄ Short-term HR variability.	HR
13	**FEAT_SD2**	⋄ Long-term HR variability.	HR
14	**FEAT_SCT**	⋄ Vector norm from the Poincaré plot centroid.	HR
15	**FEAT_SAR**	⋄ Ellipse area based on *SD*1 and *SD*2.	HR

**Table 2:** Description of each execution test according to preprocessing, processing and feature extraction.

Tests	Preprocessing		Processing, Feature Extraction and Recognition					Data		
	Detrend	Outliers	FE*	SVD	CC*	*φ_j_*(*v_j_*(*n*))	Optmization	HR	GSR	EEG
Test 1	−	−	−	−	−	sigmoid	‘sgd’	×	×	×
Test 2	−	−	−	−	−	sigmoid	‘adam’	×	×	×
Test 3	×	×	×	−	×	ReLU	‘adam’	×	×	×
Test 4	×	×	×	−	×	sigmoid	‘sgd’	×	×	×
Test 5	×	×	×	−	×	sigmoid	‘adam’	×	×	×
Test 6	×	×	×	−	×	ReLU	‘sgd’	×	×	×
Test 7	×	×	×	−	×	ReLU	‘adam’	−	×	×
Test 8	×	×	×	−	×	sigmoid	‘sgd’	−	×	×
Test 9	×	×	×	−	×	sigmoid	‘adam’	−	×	×
Test 10	×	×	×	−	×	ReLU	‘sgd’	−	×	×
Test 11	×	×	×	−	×	ReLU	‘adam’	×	−	×
Test 12	×	×	×	−	×	sigmoid	‘sgd’	×	−	×
Test 13	×	×	×	−	×	sigmoid	‘adam’	×	−	×
Test 14	×	×	×	−	×	ReLU	‘sgd’	×	−	×
Test 15	×	×	×	−	×	ReLU	‘adam’	×	×	−
Test 16	×	×	×	−	×	sigmoid	‘sgd’	×	×	−
Test 17	×	×	×	−	×	sigmoid	‘adam’	×	×	−
Test 18	×	×	×	−	×	ReLU	‘sgd’	×	×	−

CC*: Column Centering—data centering for each data. FE*: Feature Extraction—select all features for each data.

**Table 3:** Description of each execution test according to preprocessing, processing and feature selection.

Tests	Preprocessing		Processing, Feature Extraction and Recognition					Data		
	Detrend	Outliers	*FE*	SVD	*CC*	*φ_j_*(*v_j_*(*n*))	Optmization	HR	GSR	EEG
**Test 19**	×	×	×	×	×	ReLU	‘adam’	×	×	×
**Test 20**	×	×	×	×	×	sigmoid	‘sgd’	×	×	×
**Test 21**	×	×	×	×	×	sigmoid	‘adam’	×	×	×
**Test 22**	×	×	×	×	×	ReLU	‘sgd’	×	×	×
**Test 23**	×	×	×	×	×	ReLU	‘adam’	−	×	×
**Test 24**	×	×	×	×	×	sigmoid	‘sgd’	−	×	×
**Test 25**	×	×	×	×	×	sigmoid	‘adam’	−	×	×
**Test 26**	×	×	×	×	×	ReLU	‘sgd’	−	×	×
**Test 27**	×	×	×	×	×	ReLU	‘adam’	×	−	×
**Test 28**	×	×	×	×	×	sigmoid	‘sgd’	×	−	×
**Test 29**	×	×	×	×	×	sigmoid	‘adam’	×	−	×
**Test 30**	×	×	×	×	×	ReLU	‘sgd’	×	−	×
**Test 31**	×	×	×	×	×	ReLU	‘adam’	×	×	−
**Test 32**	×	×	×	×	×	sigmoid	‘sgd’	×	×	−
**Test 33**	×	×	×	×	×	sigmoid	‘adam’	×	×	−
**Test 34**	×	×	×	×	×	ReLU	‘sgd’	×	×	−

**Table 4:** Emotion recognition results tests 1 and 2. ANN with 6 × 10^3^ train epochs and raw data (no feature extraction).

	**Test 1—Emotion Recognition + RTOR**										
	***φ_j_*(*v_j_*(*n*)) = *Sigmoid*, opt = ‘sgd’, *N_h_* = 10 × 2, *N_o_* = 5**										
**DS**	**Happy**		**Sad**		**Angry**		**Surprised**		**Scared**		**Match**
	***RMSE***	***MAE***	***RMSE***	***MAE***	***RMSE***	***MAE***	***RMSE***	***MAE***	***RMSE***	***MAE***	**Accuracy (%)**
**RC1**	3.64	0.06	4.14	0.06	3.83	0.05	3.43	0.06	5.08	0.08	50.50 (1854/3671)
**RC2**	4.34	0.06	5.72	0.07	3.59	0.05	3.84	0.06	5.88	0.09	82.13 (3488/4247)
**RC3**	3.88	0.05	9.58	0.11	3.78	0.06	3.62	0.06	5.57	0.09	58.83 (2342/3981)
**GC1**	5.68	0.09	8.46	0.13	7.34	0.11	4.58	0.07	5.79	0.09	23.55 (961/4081)
**GC3**	5.63	0.09	7.45	0.11	7.41	0.11	5.42	0.08	5.84	0.09	99.65 (4240/4255)
**LS1**	5.70	0.08	6.22	0.08	3.46	0.04	5.18	0.07	6.20	0.08	22.49 (1250/5558)
**LS2**	5.52	0.09	3.68	0.05	2.93	0.04	5.04	0.08	5.42	0.08	69.43 (2844/4096)
**VC1**	3.98	0.08	3.38	0.06	4.40	0.08	3.43	0.07	4.79	0.08	15.63 (408/2611)
**VC2**	3.76	0.08	3.89	0.08	4.27	0.09	2.78	0.06	2.53	0.05	30.12 (615/2042)
**CR1**	4.46	0.07	17.54	0.24	5.00	0.06	3.58	0.06	1.64	0.02	92.47 (3697/3998)
**CR3**	1.69	0.08	3.66	0.15	1.16	0.04	1.39	0.07	1.28	0.05	74.18 (339/457)
**CLX**	4.45	0.16	1.00	0.04	1.73	0.07	1.47	0.06	0.54	0.02	0.00 (0/518)
**CL3**	3.27	0.04	3.07	0.04	5.58	0.07	5.39	0.08	3.76	0.05	15.57 (735/4722)
	4.31 ± 1.11	0.08 ± 0.02	5.98 ± 4.05	0.09 ± 0.05	4.19 ± 1.77	0.07 ± 0.02	3.78 ± 1.29	0.07 ± 0.00	4.18 ± 1.92	0.07 ± 0.02	48.81 ± 31.67
	**Test 2—Emotion Recognition + RTOR**										
	***φ_j_*(*v_j_*(*n*)) = *Sigmoid*, opt = ‘Adam’, *N_h_* = 10 × 2, *N_o_* = 5**										
**DS**	**Happy**		**Sad**		**Angry**		**Surprised**		**Scared**		**Match**
	***RMSE***	***MAE***	***RMSE***	***MAE***	***RMSE***	***MAE***	***RMSE***	***MAE***	***RMSE***	***MAE***	**Accuracy (%)**
**RC1**	1.19	0.02	5.44	0.08	3.63	0.05	0.79	0.01	1.76	0.03	50.50 (1854/3671)
**RC2**	1.26	0.02	5.77	0.07	2.41	0.03	1.02	0.01	1.73	0.03	82.13 (3488/4247)
**RC3**	4.96	0.06	9.14	0.12	4.81	0.07	0.73	0.01	3.44	0.05	58.83 (2342/3981)
**GC1**	0.64	0.01	3.97	0.06	2.96	0.05	0.64	0.01	0.74	0.01	23.55 (961/4081)
**GC3**	0.63	0.01	3.69	0.06	3.34	0.05	0.34	0.01	0.84	0.01	99.65 (4240/4255)
**LS1**	0.69	0.01	1.71	0.02	3.47	0.04	0.97	0.01	0.35	0.00	22.49 (1250/5558)
**LS2**	0.49	0.01	3.63	0.04	2.99	0.04	0.44	0.01	0.27	0.00	69.43 (2844/4096)
**VC1**	0.81	0.01	2.20	0.04	2.39	0.04	0.39	0.01	7.67	0.13	15.63 (408/2611)
**VC2**	0.28	0.01	1.76	0.03	1.07	0.02	0.96	0.02	4.68	0.09	30.12 (615/2042)
**CR1**	2.48	0.04	16.56	0.23	5.05	0.07	0.67	0.01	1.93	0.03	92.47 (3697/3998)
**CR3**	1.03	0.05	2.83	0.12	1.34	0.05	0.48	0.02	1.75	0.06	74.18 (339/457)
**CLX**	5.66	0.22	0.92	0.04	2.27	0.09	0.32	0.01	1.08	0.05	0.00 (0/518)
**CL3**	3.49	0.04	4.57	0.05	9.82	0.13	0.20	0.00	2.24	0.03	15.57 (735/4722)
	1.82 ± 1.71	0.04 ± 0.05	4.78 ± 3.98	0.07 ± 0.05	3.50 ± 2.13	0.06 ± 0.02	0.61 ± 0.26	0.01 ± 0.00	2.19 ± 1.97	0.04 ± 0.03	48.81 ± 31.67

**Table 5:** Emotion recognition results tests 11 and 12. ANN with 6 × 10^3^ train epochs and input data with feature extraction.

	**Test 11—Emotion Recognition + RTOR [HR+EEG]**										
	***φ_j_*(*v_j_*(*n*)) = *ReLU*, opt = ‘Adam’, *N_h_* = 83 × 2, *N_o_* = 5**										
**DS**	**Happy**		**Sad**		**Angry**		**Surprised**		**Scared**		**Match**
	***RMSE***	***MAE***	***RMSE***	***MAE***	***RMSE***	***MAE***	***RMSE***	***MAE***	***RMSE***	***MAE***	**Accuracy (%)**
**RC1**	0.25	0.02	0.46	0.04	0.60	0.06	0.12	0.01	0.82	0.09	22.38 (15/67)
**RC2**	0.34	0.03	0.90	0.08	0.81	0.08	0.14	0.01	0.30	0.03	34.61 (27/78)
**RC3**	0.83	0.08	1.67	0.14	0.43	0.04	0.12	0.01	0.51	0.04	38.35 (28/73)
**GC1**	0.71	0.07	1.28	0.13	0.74	0.08	0.14	0.01	0.22	0.02	21.33 (16/75)
**GC3**	0.29	0.03	0.64	0.05	0.42	0.04	0.18	0.02	0.04	0.00	65.38 (51/78)
**LS1**	0.19	0.01	1.26	0.11	0.43	0.03	0.11	0.01	0.16	0.01	25.54 (26/102)
**LS2**	0.32	0.03	0.43	0.04	0.35	0.03	0.16	0.02	0.23	0.02	42.66 (32/75)
**VC1**	0.09	0.01	0.35	0.04	0.42	0.05	0.05	0.01	1.09	0.14	16.66 (8/48)
**VC2**	0.20	0.03	0.61	0.08	0.65	0.10	0.08	0.01	0.57	0.08	21.05 (8/38)
**CR1**	0.15	0.01	2.53	0.26	0.76	0.07	0.12	0.01	0.51	0.06	47.94 (35/73)
**CR3**	0.10	0.02	0.65	0.20	0.40	0.12	0.03	0.01	0.27	0.07	44.44 (4/9)
**CLX**	0.76	0.21	0.31	0.08	0.43	0.12	0.05	0.01	0.18	0.05	0.00 (0/10)
**CL3**	0.40	0.04	1.10	0.09	0.96	0.09	0.19	0.02	0.26	0.02	19.76 (17/86)
	0.36 ± 0.24	0.05 ± 0.05	0.94 ± 0.60	0.10 ± 0.06	0.57 ± 0.18	0.07 ± 0.03	0.11 ± 0.04	0.01 ± 0.00	0.40 ± 0.28	0.05 ± 0.03	30.78 ± 16.27
	**Test 12—Emotion Recognition + RTOR [HR+EEG]**										
	***φ_j_*(*v_j_*(*n*)) = *Sigmoid*, opt = ‘sgd’, *N_h_* = 83 × 2, *N_o_* = 5**										
**DS**	**Happy**		**Sad**		**Angry**		**Surprised**		**Scared**		**Match**
	***RMSE***	***MAE***	***RMSE***	***MAE***	***RMSE***	***MAE***	***RMSE***	***MAE***	***RMSE***	***MAE***	**Accuracy (%)**
**RC1**	0.17	0.02	0.42	0.04	0.50	0.04	0.11	0.01	0.31	0.04	53.73 (36/67)
**RC2**	0.23	0.02	0.82	0.07	0.29	0.03	0.13	0.01	0.39	0.04	82.05 (64/78
**RC3**	0.67	0.06	1.37	0.11	0.29	0.03	0.11	0.01	0.35	0.04	57.53 (42/73)
**GC1**	0.36	0.04	0.96	0.11	0.69	0.08	0.21	0.02	0.38	0.04	22.66 (17/75)
**GC3**	0.35	0.04	0.82	0.09	0.70	0.08	0.32	0.04	0.39	0.04	100.00 (78/78)
**LS1**	0.31	0.03	0.64	0.06	0.30	0.02	0.23	0.02	0.37	0.04	22.54 (23/102)
**LS2**	0.34	0.04	0.38	0.04	0.22	0.02	0.27	0.03	0.33	0.04	68.00 (51/75)
**VC1**	0.22	0.03	0.31	0.04	0.35	0.05	0.14	0.02	0.90	0.11	16.66 (8/48)
**VC2**	0.23	0.04	0.42	0.06	0.37	0.06	0.10	0.02	0.47	0.06	28.94 (11/38)
**CR1**	0.22	0.02	2.58	0.26	0.90	0.09	0.10	0.01	0.27	0.03	93.15 (68/73)
**CR3**	0.10	0.03	0.55	0.15	0.17	0.05	0.05	0.02	0.26	0.07	77.77 (7/9)
**CLX**	0.71	0.20	0.06	0.02	0.36	0.10	0.06	0.02	0.10	0.03	0.00 (0/10)
**CL3**	0.28	0.02	0.39	0.03	1.07	0.10	0.29	0.03	0.16	0.01	15.11 (13/86)
	0.32 ± 0.17	0.05 ± 0.04	0.75 ± 0.61	0.08 ± 0.06	0.48 ± 0.26	0.06 ± 0.02	0.16 ± 0.08	0.02 ± 0.00	0.36 ± 0.18	0.05 ± 0.02	49.09 ± 32.00
